# Associations of Maternal and Neonatal Serum Trace Element Concentrations with Neonatal Birth Weight

**DOI:** 10.1371/journal.pone.0075627

**Published:** 2013-09-27

**Authors:** Shinya Tsuzuki, Nao Morimoto, Shinichi Hosokawa, Takeji Matsushita

**Affiliations:** Department of Pediatrics, National Center for Global Health and Medicine, Tokyo, Japan; Emory University School of Medicine, United States of America

## Abstract

**Background:**

Trace elements play important nutritional roles in neonates. Our objective was to examine whether there are differences in maternal/neonatal serum trace element concentrations between mature infants and premature infants.

**Methods:**

During 2012, 44 infants born at National Center for Global Health and Medicine, Tokyo, Japan, were enrolled. Serum samples were collected to measure serum iron, zinc, copper, and selenium concentrations 5 days after birth. Maternal serum samples were obtained before delivery and cord blood was taken at delivery to measure the same trace elements. We compared the results between term group whose birth weight were ≥2500 g and gestational age were ≥37 weeks and premature group whose birth weight were <2500 g or gestational age were <37 weeks. Variables significantly different between two groups were included in linear regression models to identify significant predictors of birth weight. Values of *P*<0.05 were considered statistically significant.

**Results:**

Serum selenium concentrations were lower in premature group than in term group (43.3±7.0 µg/L vs. 52.0±8.9 µg/L, *P* = 0.001). Maternal serum selenium concentrations were also significantly lower in the mothers of premature group than in the mothers of term group (79.3±19.3 µg/L vs. 94.1±18.1 µg/L, *P* = 0.032). There were no significant differences in neonatal or maternal iron, zinc, or copper concentrations between two groups. Multivariate linear regression analysis showed that, except for gestational age, only maternal serum selenium was significantly associated with birth weight (*P* = 0.015).

**Conclusions:**

Serum selenium concentrations were lower in premature group and their mothers compared with the term group. The maternal serum selenium concentration was positively correlated with birth weight. These results suggest that maternal serum selenium concentration may influence neonatal birth weight.

## Introduction

Although trace elements are an essential nutritional component of humans, their specific roles are still poorly understood, especially in the perinatal period. Several micronutrients are thought to affect the growth of fetuses and infants. Some studies have also examined the effectiveness of micronutrient supplementation during pregnancy. One study in Nepal found that a multiple micronutrient supplement taken during pregnancy resulted in increases in infant weight that were sustained until ≥2 years of age [1]. In Peru, the infants of women who received an iron (Fe), folic acid, and zinc (Zn) supplement during pregnancy were heavier at 4 months of age than children of women who received an Fe and folic acid supplement [2]. However, an antenatal Zn supplementation trial conducted in Bangladesh found no differences in infant weight through to 6 months of age [3].

Some studies have examined whether micronutrient interventions during pregnancy can improve birth weight and reduce early infant mortality in developing countries [4]–[6], and a meta-analysis concluded that multiple micronutrient supplementation may provide a beneficial impact over a traditionally recommended iron and folic acid supplement [7]. However, these studies were conducted in developing countries and there is limited research in developed countries. In addition, the effects of individual elements are unknown because most of the prior studies used multi-vitamin, multi-micronutrient supplements.

In Japan, very few studies have examined the effects of individual trace elements. Kon et al. reported a correlation between serum iron concentrations and developmental outcomes in preterm infants [8]. However, Sugiura et al. raised concern over a possible developmental delay in children with atopic dermatitis who had been treated with Zn [9]. Nevertheless, very little is known about the effects of micronutrients on children, especially on neonates. Therefore, we examined the possible associations between four trace elements and birth weight in Japanese infants.

## Materials and Methods

### Ethical Statement

This study was approved by the Ethics Committee of the National Center for Global Health and Medicine (NCGM), Tokyo, Japan. Participating women were interviewed and tested after providing written informed consent.

### Settings and Participants

The study population consisted of 44 neonates who were born at the NGCM (one of local perinatal center in Tokyo, in which 358 neonates were delivered in 2012) between January and December 2012, forming a retrospective cohort. 44 (27 boys, 17 girls) out of 358 neonates (176 boys, 182 girls) were enrolled. Because our study protocol required maternal serum samples and aimed to avoid unnecessary blood drawing with the sole purpose of research, only mothers whose blood samples had already been obtained before delivery for some other reasons were regarded eligible. Blood samples were collected from the neonates at 5 days of age to measure serum Fe, Zn, copper (Cu), and selenium (Se) concentrations because all neonates born in Japan are performed blood testing for newborn screening at 5 days of age. Maternal serum samples were obtained before delivery (usually on the day of admission) and cord blood was obtained at delivery. These samples were also used to measure the concentrations of Fe, Zn, Cu, and Se. The mothers underwent medical interviews on admission to determine their age, body mass index (BMI) before pregnancy and before delivery, medical history, smoking status, and alcohol consumption. Birth weight and gestational age at delivery were obtained retrospectively from the medical records. Gestational age was calculated by last menstrual period. The infants and their mothers were divided into a term group (whose birth weight were ≥2500 g and gestational age were ≥37 weeks) or a premature group (whose birth weight were <2500 g or gestational age were <37 weeks).

### Statistical Analysis

Statistical analyses were performed using SPSS software version 20.0. Fisher's exact test was used to compare categorical variables between the mature and LBWI groups. Student's *t* test or the Mann–Whitney U test was used to compare continuous variables between the two groups. Variables that differed between the two groups at *P*<0.10 were included in univariate and stepwise multivariate linear regression models to identify independent predictors of birth weight. To exclude the possibility of a false-positive association, multicollinearity of predictors was assessed using the variance inflation factor (VIF), and predictors with VIF>5 were removed from the final model. For linear regression, values of *P*<0.05 were considered statistically significant.

## Results

Of the 44 infants, 14 were classified into premature group. 8 were <2500 g, 13 were <37 weeks gestational age and 2 were intrauterine growth retardation (IUGR). The mean ± standard deviation gestational age was 35.6±2.3 weeks in the premature group and 39.5±1.4 weeks in the term group. The mean birth weight was 2388.1±465.0 g in the premature group and 3043.4±321.2 g in the term group. There were no significant differences in the background characteristics of the mothers. (Table 1).

**Table 1:** Baseline characteristics of LBWIs and term infants.

Variable	Premature	Term	*P* value
**Males/total**	11/14	16/30	0.509^1^
**GA (weeks)**	35.6±2.3	39.5±1.4	<0.001^2^
**BW (g)**	2388.1±465.0	3043.4±321.2	<0.001^2^
**IUGR**	2/14	0/30	NA
**Breastfeeding**	12/14	29/30	0.234^2^
**BMI before pregnancy (kg/m^2^)**	21.4±3.3	20.5±2.6	0.284^2^
**BMI during pregnancy (kg/m^2^)**	24.7±4.1	24.9±2.7	0.946^2^
**Maternal age (years)**	34.6±4.9	35.3±4.6	0.604^2^
**Maternal smoking, yes (%)**	0	7.4	NA
**Maternal alcohol consumption, yes (%)**	0	7.4	NA
**GBS status, yes (%)**	0	26.9	0.155^1^

Results are means ± standard deviation or %.

GA, gestational age; BW, body weight; IUGR, intrauterine growth retardation; BMI, body mass index; GBS, group B streptococcus detected from vaginal smear culture; NA, not available.

1 Fisher's exact test.

2 Mann–Whitney U test.

The neonatal serum Se concentration on day 5 was significantly different between premature and term groups (43.3±7.0 µg/L vs. 52.0±8.9 µg/L, *P* = 0.001). However, there were no significant differences in Fe, Zn, or Cu between these infants. Maternal serum Se concentrations were also significantly different between these two groups (79.3±19.3 µg/L vs. 94.1±18.1 µg/L, *P* = 0.032). The cord blood of term group had slightly higher Se and Cu concentrations compared with cord blood of premature group. The cord blood Se concentration was 52.8±9.7 µg/L in premature group and 59.4±10.0 µg/L in term group (*P* = 0.071). The cord blood Cu concentration was 28.3±10.0 µg/L in premature group and 36.4±11.9 µg/L in term group (*P* = 0.056). (Tables 2, 3, 4)

**Table 2:** Comparison of neonatal serum Fe, Zn, Cu, and Se concentrations at 5 days of age between LBWIs and term infants.

	Premature	Term	*P* value^1^
**Fe (µg/dl)**	92.0±35.4	109.3±48.6	0.263
**Zn (µg/dl)**	84.7±19.7	75.8±12.0	0.279
**Cu (µg/dl)**	43.4±14.9	54.4±17.3	0.126
**Se (µg/L)**	43.3±7.0	52.0±8.9	0.001

Results are means ± standard deviation.

Fe, iron; Zn, zinc; Cu, copper; Se, selenium.

1 Mann–Whitney U test.

**Table 3:** Comparison of maternal serum Fe, Zn, Cu, and Se concentrations before delivery between LBWIs and term infants.

	Premature	Term	*P* value^1^
**Fe (µg/dl)**	56.5±42.3	62.6±38.2	0.685
**Zn (µg/dl)**	48.8±16.0	54.0±10.9	0.332
**Cu (µg/dl)**	218.4±49.8	229.2±38.7	0.524
**Se (µg/L)**	79.3±19.3	94.1±18.1	0.032

Results are means ± standard deviation.

Fe, iron; Zn, zinc; Cu, copper; Se, selenium.

1 Student's *t* test.

**Table 4:** Comparison of cord blood Fe, Zn, Cu, and Se concentrations after delivery between LBWIs and term infants.

	Premature	Term	*P* value^1^
**Fe (µg/dl)**	153.6±59.9	173.4±59.4	0.350
**Zn (µg/dl)**	89.0±14.3	85.7±16.4	0.553
**Cu (µg/dl)**	28.3±10.0	36.4±11.9	0.056
**Se (µg/L)**	52.8±9.7	59.4±10.0	0.071

Results are means ± standard deviation.

Fe, iron; Zn, zinc; Cu, copper; Se, selenium.

1 Student's *t* test.

Stepwise linear regression analysis was performed, in which the insignificant independent variables were removed one by one. Other than gestational age, only one variable, maternal Se, was independently associated with birth weight. The regression coefficient was 10.985 (95% confidence interval: 2.391, 19.578; *P* = 0.015). The results of the univariate and multivariate regression analyses are presented in Table 5.

**Table 5:** Results of univariate and multivariate linear regression analysis.

Variable	Univariate analysis		Multivariate analysis	
	*β* (95% CI)	*P* value	*β* (95% CI)	*P* value
**GA**	12.599 (6.019, 19.179)	<0.001	11.877 (4.541, 19.212)	**0.003**
**NeoSe**	29.555 (15.689, 43.421)	<0.001	17.783 (−5.590, 41.155)	0.128
**MatSe**	11.193 (3.828, 18.558)	0.004	10.985 (2.391, 19.578)	**0.015**
**UmbCu**	9.591 (−3.101, 22.284)	0.134	−0.044 (−14.097, 14.008)	0.995
**UmbSe**	12.801 (−0.342, 25.944)	0.056	−14.524 (−33.134, 4.085)	0.119

***β***, regression coefficient; CI, confidence interval; GA, gestational age; NeoSe, neonatal serum selenium concentration; MatSe, maternal serum selenium concentration; UmbCu, umbilical cord blood serum copper concentration; UmbSe, umbilical cord blood serum selenium concentration.

## Discussion

The results of the present study indicated that maternal serum Se concentration was an independent predictor of birth weight. In fact, some studies have reported possible effects of Se on neonates. For example, Brian et al. reported an association between serum Se and respiratory outcomes in very-low-birth-weight infants [10]. However, that study measured serum Se concentrations at 28 days of age. Therefore, it remains unclear whether Se concentrations at birth or soon after birth are associated with respiratory outcomes. Although Giménez et al. reported that LBWIs had lower serum Se concentrations [11], their study was published in Spanish, which limited dissemination of their results. Nevertheless, their study revealed a possible relationship between Se and birth weight in neonates.

In the present study, Fe, Zn, and Cu concentrations in the infant and mother were not associated with birth weight. To our knowledge, no study has demonstrated an association between serum Fe concentrations and birth weight. However, Khadam et al. reported that maternal Zn concentrations were negatively associated with birth weight [12]. Soo et al. showed that serum Cu concentrations were higher in infants weighing <1000 g than in those weighing 1000–1500 g [13]. Clearly, further studies are necessary to examine the effects, if any, of these three trace elements on birth weight.

Multiple factors, including maternal nutritional status, can influence birth weight. BMI before pregnancy is often used as a marker of the mother's nutritional status, and can affect the birth weight of infants [14], [15]. However, in our study, maternal BMI was not associated with neonatal birth weight. Maternal serum Se concentrations were not dependent on their BMI. In addition, gestational age showed no Univariate correlation with maternal Se (*β* = 0.100, *P* = 0.564). These findings suggest that maternal serum Se concentrations are a predictor of neonatal birth weight, independent of maternal nutritional status.

Our study provides further evidence for a positive association between Se concentrations and neonatal birth weight. If maternal Se concentrations are a potential risk factor for low birth weight, independent of maternal nutritional status, measuring maternal serum Se concentrations may be useful to identify LBWIs or preterm delivery, even in women with a normal BMI.

There are some limitations to our study. First, the similar characteristics of the enrolled subjects might be due to the small sample size and the study design. Our database included only 44 infants born in 2012 at a single institute. Although we tried to exclude known confounding factors that may reflect maternal nutritional status, some confounders may remain. Furthermore, we have no robust hypothesis to explain why Se concentrations can affect neonatal birth weight, and very few studies have examined the potential mechanisms. Although it can be inferred that maternal trace elements are transported through the placenta, there were no significant differences in cord blood concentrations of the four trace elements between term infants and premature infants, which may undermine our conclusion. Therefore, we think that determining the mechanism by which maternal Se affects neonatal birth weight is an important topic for future research.

Generally speaking, Se is incorporated into proteins to make selenoproteins, including the glutathione peroxidase antioxidant enzymes, thioredoxin reductases, and selenoprotein-P. Most of required Se values in which worldwide difference exist has been determined from the intake believed necessary to maximize the activity of the antioxidant glutathione peroxidase in plasma. Therefore low serum concentration of Se may be considered as a potential indicator of Se deficiency, although the level at which symptoms due to Se deficiency become present has not been obvious yet.

Hiten and Paula insisted that recurrent early pregnancy loss has been associated with reduced serum selenium concentrations compared to healthy controls in some studies [16]. They also hypothecated that reduced selenium concentration results in reduced glutathione peroxidase activity culminating in reduced antioxidant protection of biological membranes and DNA during the early stages of embryonic development. Although speculative and larger placebo-controlled randomized trials would be needed to verify their opinion, it may be worth considering antioxidant mechanism as one cause of co-linearity between maternal Se concentration and birth weights.

## Conclusions

We found that serum Se concentrations were lower in premature infants than in mature infants. Additionally, maternal serum Se concentrations were positively correlated with neonatal birth weight. However, we found no association between maternal BMI or gestational age and serum Se concentrations. These results suggest that maternal serum Se concentrations may have an effect on neonatal birth weight, and that serum Se concentrations are an independent predictor of neonatal birth weight that is independent of the mother's nutritional status. Further studies are necessary to confirm the generalizability of our results.
